# Circular RNA circCHFR downregulation protects against oxidized low-density lipoprotein-induced endothelial injury via regulation of microRNA-15b-5p/growth arrest and DNA damage inducible gamma

**DOI:** 10.1080/21655979.2022.2032967

**Published:** 2022-02-09

**Authors:** Yang Li, Bing Wang

**Affiliations:** Department of Vascular Surgery, The Fifth Affiliated Hospital of Zhengzhou University, Zhengzhou, China

**Keywords:** Atherosclerosis, circRNA, circCHFR (hsa_circ_0029589), miRNA, miR-15b-5p, GADD45G

## Abstract

Atherosclerosis is the leading cause of coronary heart disease. In recent years, circ_0029589 (circCHFR) has been found to be associated with atherosclerosis development. However, the molecular mechanism of circCHFR action in atherosclerosis development is unknown. This study was aimed to investigate the function and action mechanism of circCHFR in atherosclerosis development. An atherosclerosis cell model was created by exposing human vascular endothelial cells (HUVECs) to oxidized low-density lipoprotein. The expression of circCHFR, microRNA(miR)-15b-5p, growth arrest and DNA damage inducible gamma (GADD45G), and their associated proteins was evaluated using quantitative reverse transcription-polymerase chain reaction and Western blotting. Additionally, cell viability, apoptosis, and cytokine levels were determined using Cell Counting Kit-8 (CCK8) assay, flow cytometry, and enzyme-linked immunosorbent assay, respectively. circCHFR expression was upregulated in patients with atherosclerosis and oxidized low-density lipoprotein (ox-LDL)-exposed HUVECs, whereas miR-15b-5p expression was downregulated. circCHFR silencing significantly improved viability and reduced apoptosis of HUVECs. In addition, the pro-apoptotic protein Bax and atherosclerosis-associated cytokines (interleukin-1β, interleukin-6, and tumor necrosis factor-α) were significantly downregulated, whereas the anti-apoptotic protein Bcl-2 was upregulated. Further, we discovered that circCHFR serves as a molecular sponge of miR-15b-5p. GADD45G was found to be an important target of miR-15b-5p; miR-15b-5p mimic inhibited GADD45G expression, reduced apoptosis and proinflammatory cytokine secretion, and improved cell survival. However, these effects of miR-15b-5p on (ox-LDL) induced HUVECs were reversed with GADD45G plasmid co-transfection. In conclusion, circCHFR promotes atherosclerosis progression via the miR-15b-5p/GADD45G axis and may be an important target for atherosclerosis treatment.

## Introduction

Atherosclerosis is a chronic condition of the arterial wall, where the artery walls become narrowed with fatty substances. It is one of the most prevalent cardiovascular diseases, resulting in a high rate of morbidity and mortality [1]. The disruption of vascular smooth muscle cells (VSMCs), collagen fiber synthesis, lipid accumulation, and endothelial cell injury are associated with the progression of atherosclerosis [2]. Endothelial cell death results in the formation of blood plaques, which contributes to the development of atherosclerosis [3]. Notably, the high abundance of ox-LDL causes VSMC dysfunction and positively correlates with atherosclerosis progression [4,5]. Ox-LDL induces aberrant cell proliferation, migration, apoptosis, angiogenesis, and inflammatory response, resulting in endothelial dysregulation [5,6]. Nevertheless, the molecular mechanisms underlying ox-LDL-induced atherosclerosis remain unclear. Therefore, it is necessary to identify mechanisms underlying the pathogenesis of atherosclerosis and plausible therapeutic targets.

In recent years, progressive studies have demonstrated that circular RNAs (circRNAs) play essential roles in the development of cardiovascular disorders, including atherosclerosis [7–9]. circRNAs are an important class of noncoding RNAs that are products of non-sequential back-splicing of pre-mRNA transcripts [10,11]. circRNAs regulate critical functions of VSMCs and contribute to cellular damage and atherosclerosis progression caused by ox-LDL [12,13]. For example, hsa_circ_0000345 and hsa_circ_0003575 play roles in ox-LDL-induced VSMC proliferation and angiogenesis [14,15]. Emerging data suggest that circRNA circ_0029589 (circCHFR), originating from the checkpoint with forkhead and ring finger domains (CHFR) gene, is upregulated in ox-LDL-exposed VSMCs and plays a pivotal role in atherosclerosis development [6,16]. However, the mechanism underlying circCHFR-induced atherosclerosis requires further investigation.

Importantly, circRNAs can act as microRNA (miRNA) sponges and control the expression and function of target mRNAs via sequence complementarity [17]. In atherosclerosis, increasing evidence has indicated that circRNA/miRNA regulatory networks are associated with atherosclerosis progression [15,18].

miRNAs are a novel class of noncoding RNAs that control epigenetic processes and post-transcriptional regulation of target genes. Recently, miRNAs have been found to play critical roles in atherosclerosis development. For instance, miR-15b-5p levels are downregulated in patients with atherosclerosis and have a protective role against it [15]. Moreover, plasma miR-15b-5p levels are inversely associated with coronary atherosclerosis [19,20]. Despite the critical function of miR-15b-5p in atherosclerosis, its molecular mechanism of action remains unknown.

Additionally, cumulative research has established that miRNAs, such as miRNA-128-1-5p, regulate gene expression and function by inhibiting growth arrest and DNA damage-inducible 45 gamma (GADD45G)-mediated apoptotic signalings [21]. In addition, GADD45G plays a critical role in stress-induced growth arrest and apoptosis [22]. Therefore, it is worthwhile to explore the function of GADD45G in atherosclerosis.

Endothelial dysfunction is a precursor of cardiovascular disease, and ox-LDL is implicated in the development of atherosclerosis through directly targeting endothelial cells [23]. And ox-LDL-induced endothelial cell injury has been widely used to explore atherosclerosis *in vitro* [23–25]. In this study, we used ox-LDL-induced endothelial cells as an atherosclerosis *in vitro* model.

In this study, we hypothesized that circCHFR down-regulation protects against ox-LDL-induced endothelial injury by regulating miR-15b-5p/GADD45G. Therefore, this study was designed to explore the role of circCHFR in human vascular endothelial cells (HUVECs). We also investigated the abundance of circCHFR, miR-15b-5p, and GADD45G in ox-LDL-treated HUVECs and their involvement in atherosclerosis, as well as the mechanism by which circCHFR contributes to atherosclerosis development via the miR-15b-5p/GADD45G axis.

## Methods and materials

### Study subjects

We collected blood samples from patients with atherosclerosis (n = 30) and age-matched healthy subjects (n = 30) as the control group, from the Fifth Affiliated Hospital of Zhengzhou University with their written informed consent. The study was approved by the ethics committee of the Fifth Affiliated Hospital of Zhengzhou University.

### Cell lines and treatment

HUVECs (American Type Culture Collection, Manassas, VA, USA) were cultured in Roswell Park Memorial Institute (RPMI) 1640 medium containing 10% (v/v) fetal bovine serum (FBS) at 37°C and 5% CO_2_. To establish the atherosclerosis cell model, HUVECs were exposed to different concentrations of ox-LDL (0, 50, 100, and 150 μg/mL) for 24 h [24].

### Plasmids and transfection

To mimic or silence genes, different oligonucleotides or plasmid vectors were transfected into HUVECs, such as with control-siRNA (GenePharma, Shanghai, China), circCHFR-siRNA (GenePharma), miR-15b-5p inhibitor (GenePharma), inhibitor control (GenePharma), miR-15b-5p mimic (GenePharma), mimic control (GenePharma), GADD45G plasmid (Santa Cruz Biotechnology, Dallas, TX, USA), and control plasmid using Lipofectamine 2000 (Invitrogen, Carlsbad, CA, USA). After 24 h of incubation at 37°C, the cells were collected, and the transfection efficiency was analyzed using RT-qPCR.

### Dual luciferase reporter assay [26]

To identify the binding site between circCHFR and miR-15b-5p, the bioinformatic tool StarBase (starBase models RNA-RNA binding between circRNAs and miRNAs) was used. For dual luciferase reporter assay, the sequences of circCHFR containing the target sequence of miR-15b-5p were obtained and cloned into a pmirGLO vector (Promega Corporation) to construct the circCHFR-wild-type (WT) reporter gene vector. A circCHFR-mutated type (MUT) reporter gene vector was also constructed using the QuikChange Site-Directed Mutagenesis kit (Stratagene; Agilent Technologies, Inc.) was applied according to the manufacturer’s instructions. Then the reporter plasmids were transfected into HUVECs with 50 ng of Renilla luciferase reporter plasmid using Lipofectamine 2000. After 24 h of incubation, the luciferase densities were determined using a dual luciferase assay kit (Promega, Madison, WI, USA), and Renilla luciferase activity was used as an internal control.

The binding sites between GADD45G and miR-15b-5p was predicted by TargetScan and confirmed by dual luciferase reporter assay as described above.

### RNA isolation and RT-qPCR analysis

HUVECs were plated at a density of 5 × 10^6^ cells/well in a six-well plate. Total RNA was extracted using FastPure RNA Isolation Kit (Biotrend), and complementary DNA was synthesized using PrimeScript^TM^ RT Master Mix (Takara Bio). qPCR was performed using a SYBR Green PCR kit (Applied Biosystems) on a QuantStudio5 detection system with duplicate samples. Thermocycling conditions used for qPCR were as follows: Initial denaturation at 95°C for 5 min; followed by 38 cycles of 15 sec at 95°C, 1 min at 60°C and 30 sec at 72°C; and a final extension for 10 min at 72°C. Relative gene expression was determined using the 2^−ΔΔCt^ technique [27], with GAPDH or U6 as the housekeeping genes. Primer sequences were listed as following:

GAPDH forward, 5′-CATCATCCCTGCCTCTACTGG-3′;

reverse, 5′-GTGGGTGTCGCTGTTGAAGTC-3′;

GADD45G forward, 5′-CAGATCCATTTTACGCTGATCCA-3′;

reverse, 5′-TCCTCGCAAAACAGGCTGAG-3′;

Bcl-2 forward, 5′-AGGATTGTGGCCTTCTTTGAG-3′;

reverse, 5′-AGCCAGGAGAAATCAAACAGAG-3′;

Bax forward, 5′-TCTGAGCAGATCATGAAGACAGG-3′;

reverse, 5′-ATCCTCTGCAGCTCCATGTTAC-3′;

U6 S, 5′-GGAACGATACAGAGAAGATTAGC-3′;

Stem-loop-R, 5′-CTCAACTGGTGTCGTGGAGTC-3′;

circCHFR forward, 5′- CTTCCAGCCCATGCCCGACCGG-3′;

reverse, 5′- CAGAAGGCAGGCGGCGCAG-3′;

miR-15b-5p forward, 5’-UAGCAGCACAUCAUGGUUUACA-3′;

reverse, 5′-CTCAACTGGTGTCGTGGA-3′.

### RNA pull-down assay [28]

For the RNA pull-down assay, biotin-tagged circCHFR (bio-circCHFR) and the corresponding negative control (bio-NC) were transfected into HUVECs. After 48 h of transfection, cells were treated with lysis buffer for 10 min. Each binding tube was supplemented with M-280 streptavidin magnetic beads and incubated for 4 h. The beads were then rinsed six times with RNase-free BSA, and TRIzol (Thermo Fisher Scientific, Inc.) was used to purify the bound RNA following the manufacturer’s protocol. The retrieved supernatant was analyzed by qRT-PCR to detect miR-15b-5p expression.

### Cytotoxicity assay [29]

Cell cytotoxicity was evaluated using the Cell Counting Kit-8 (CCK8) assay. Briefly, HUVECs (1 × 10^4^ cells/well) were plated in a 96-well plate and incubated for 24 h. Then, the cells were exposed to ox-LDL and sampled at 0, 24, 48, and 72 h. After sampling, 10 μL CCK8 solution was added to each well and incubated at 37°C for 1 h. Finally, the absorbance was determined at 490 nm wavelength using a spectrophotometer.

### Flow cytometry [30]

An annexin V-FITC Apoptosis Detection Kit (Beyotime, Shanghai, China) was used to analyze early apoptosis. After being exposed to ox-LDL, HUVECs were collected from the supernatants and rinsed with phosphate-buffered saline (PBS). Next, the cells were stained with annexin V-FITC and PI. Apoptosis was determined using a flow cytometer (BD, USA) and analyzed using FlowJo software version 10.8.1.

### Western blotting [31]

Cultured cells were collected, and RIPA lysis buffer (Solarbio) containing protease and phosphatase inhibitors was used for protein extraction. Following protein extraction, a BCA protein assay kit (Tanon, Shanghai, China) was used to quantify protein concentration, and then approximately 40 g of protein samples was separated using sodium dodecyl sulfate-polyacrylamide gel electrophoresis. They were then transferred onto polyvinylidene difluoride membranes. Subsequently, the membranes were incubated with 5% nonfat milk to block nonspecific binding, followed by incubating with primary antibodies: Bcl-2 (1:1,000 dilution, cat. no. ab32124; Abcam, Cambridge, MA, USA), Bax (1:1,000 dilution, cat. no. ab182734; Abcam), GADD45G (1:1,000 dilution, cat. no. sc-393,261; Santa Cruz Biotechnology), and GAPDH (1:2,500 dilution, cat. no. ab9485; Abcam) for 24 h at 4°C. The membranes were then washed with PBST and incubated with secondary antibodies (1:2,000 dilution, cat. no. ab7090/ab7068, Abcam) for 2 h to allow conjugation with primary antibodies. After washing the membranes, an ECL substrate kit (Abcam) was used to analyze protein signals. Protein signal data were analyzed using ImageJ software version 1.8.0 (NIH, Bethesda, MD, USA).

### Enzyme-linked immunosorbent assay (ELISA) [32]

The cells were harvested and centrifuged at 1000 × *g* for 10 min at 4°C. Subsequently, the cells were discarded, supernatants were collected, and specific ELISA kits were used to evaluate TNF-α (cat. no. PT518; Beyotime), IL-6 (cat. no. PI330; Beyotime), and IL-1β (cat. no. PI305; Beyotime) levels in the supernatants, following the manufacturer’s instructions.

### Statistical analysis

GraphPad Prism 6 (GraphPad, USA) was used to conduct the statistical analysis. The data were presented as Mean ± Standard Deviation. To compare the differences between groups, the Student’s t-test or ANOVA with Tukey’s post hoc test were utilized. The data were considered significant at *P* < 0.05.

## Results

### Expression profile of circCHFR and miR-15b-5p in patients with atherosclerosis

**Figure 1.**
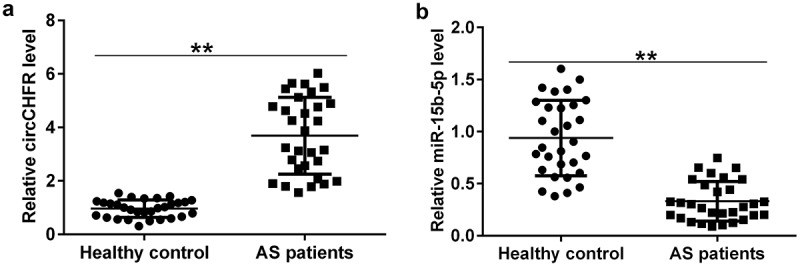
Serum levels of circCHFR and miR-15b-5p in atherosclerosis patients.

To understand the levels of circCHFR and miR-15b-5p in patients with atherosclerosis, we collected 60 blood samples, including 30 from patients with atherosclerosis and 30 from healthy subjects as the control group. The expression levels of circCHFR and miR-15b-5p were determined using qRT-PCR. Compared to the healthy subjects, the abundance of circCHFR in the serum of atherosclerosis patients was significantly upregulated (Figure 1a), while the level of miR-15b-5p was downregulated (Figure 1b).

The expression of (A) circCHFR and (B) miR-15b-5b in the serum samples from 30 patients with atherosclerosis and 30 age-matched healthy subjects was detected by qRT-PCR (n = 30). All experiments were repeated three times. **p < 0.01.

### Relationship between circCHFR and miR-15b-5p

**Figure 2.**
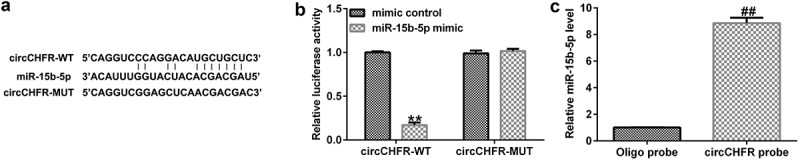
Relationship between circCHFR and miR-15b-5p.

To identify the binding site between circCHFR and miR-15b-5p (Figure 2a), the bioinformatic tool StarBase (starBase models RNA-RNA binding between circRNAs and miRNAs) was used. There are more than 100 miRNAs that have binding sites with circCHFR, including miR-15b-5p. Furthermore, we verified the binding site between circCHFR and miR-15b-5p using a dual fluorescent protein reporter system (Figure 2b) and RNA pull-down assay (Figure 2c), which suggested the molecular interaction between circCHFR and miR-15b-5p in HUVEC cultures.

(A) Bioinformatic tool StarBase depicting the relationship between circCHFR and miR-15b-5p; Verification of binding site by (B) Dual fluorescence assay and (C) RNA pull down technique. All experiments were repeated three times. **p < 0.01 vs. mimic control; ##*p* < 0.01 vs. Oligo probe.

### CircCHFR and miR-15b-5p expression in ox-LDL-treated HUVECs

**Figure 3.**
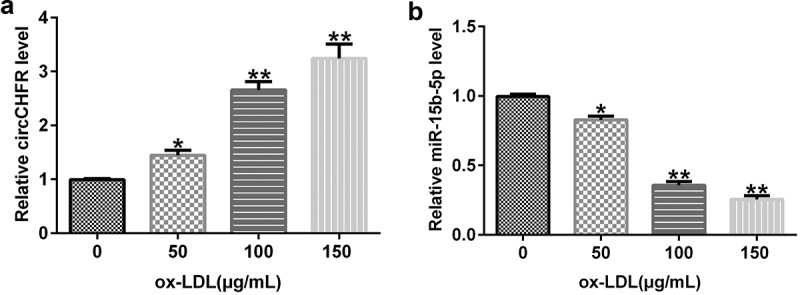
Expression profile of circCHFR and miR-15b-5p in ox-LDL-induced HUVECs.

To examine the impact of ox-LDL treatment on HUVECs, HUVECs were treated with varying amounts of ox-LDL (0, 50, 100, and 150 μg/mL) for 24 h, followed by qRT-PCR to determine the expression of circCHFR and miR-15b-5p. In comparison with that in the untreated group, ox-LDL dose-dependently boosted the expression of circCHFR in HUVECs (Figure 3a), whereas miR-15b-5p was considerably downregulated (Figure 3b).

Expression of circCHFR (A) and miR-15b-5p (B) was detected by qRT-PCR in HUVECs treated with varying levels of ox-LDL (0, 50, 100, and 150 μg/mL) for 24 h. All experiments were repeated three times. *, **p < 0.05, 0.01 vs. Control.

### Regulatory effect of circCHFR on miR-15b-5p expression in HUVECs

**Figure 4.**
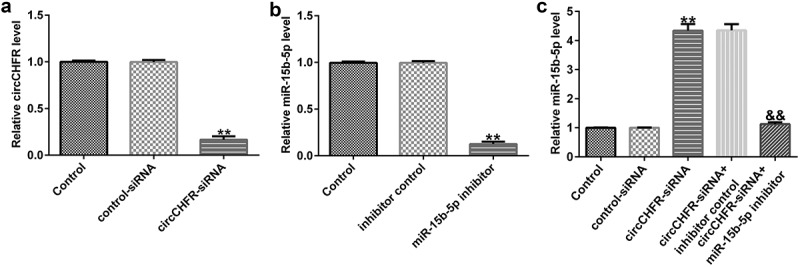
CircCHFR negatively regulates miR-15b-5p expression in HUVECs.

To understand the impact of circCHFR on miR-15b-5b, control-siRNA or circCHF-siRNA, inhibitor control or miR-15b-5p inhibitor, circCHFR-siRNA+inhibitor control, or circCHFR-siRNA+miR-15B-5p inhibitor plasmids were transfected into HUVECs. Transfection effectiveness was determined by qRT-PCR. circCHFR-siRNA greatly reduced the expression of circCHFR in HUVECs (Figure 4a). Furthermore, the miR-15b-5p inhibitor significantly reduced the expression of miR-15b-5p (Figure 4b). Interestingly, circCHFR-siRNA greatly increased miR-15b-5p expression in HUVECs. However, co-transfection with the miR-15b-5p inhibitor starkly reversed this effect (Figure 4c). Therefore, circCHFR negatively regulated miR-15b-5p expression.

(A) Gene expression of circCHFR-siRNA in HUVECs transfected with control-siRNA and circCHFR-siRNA was detected using RT-qPCR; (B) Gene expression of miR-15b-5p in HUVECs transfected with inhibitor control and miR-15b-5p inhibitor was detected using RT-qPCR; (C) Gene expression of miR-15b-5p in HUVECs transfected with control-siRNA, circCHFR-siRNA, circCHFR-siRNA+inhibitor control and circCHFR-siRNA+miR-15b-5p inhibitor was detected using RT-qPCR. All experiments were repeated three times. **p < 0.01 vs. Control-siRNA; ##*p* < 0.01 vs. inhibitor control; &&*p* < 0.01 vs. circCHFR-siRNA+inhibitor control.

### circCHFR gene silencing protects against HUVEC injury via miR-15b-5p upregulation

**Figure 5.**
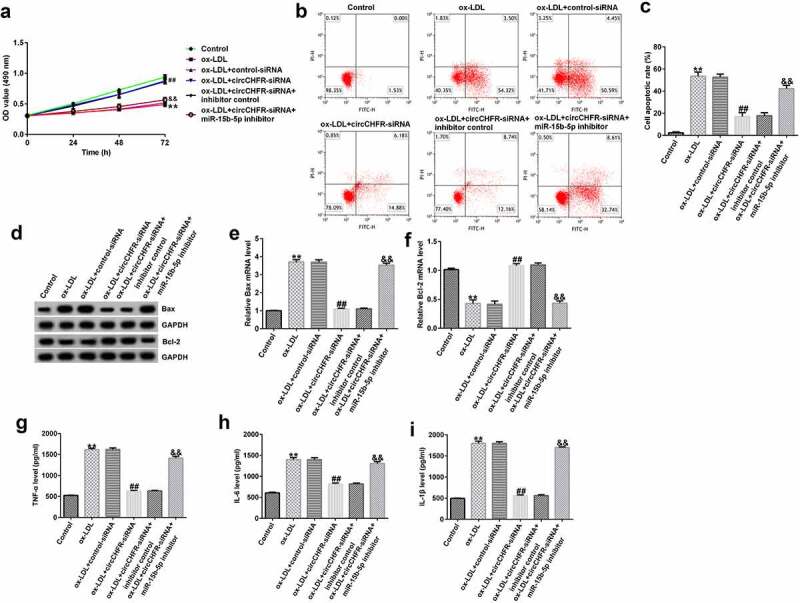
Protective role of circCHFR-siRNA in ox-LDL-exposed HUVECs.

To investigate the role of circCHFR and miR-15b-5p in ox-LDL induced HUVEC injury, after transfection with inhibitor control or miR-15b-5p inhibitor, control-siRNA or circCHFR-siRNA, and circCHFR-siRNA+inhibitor control or circCHFR-siRNA+miR-15b-5p inhibitor, HUVECs were stimulated for 24 h with 100 μg/mL ox-LDL. Following ox-LDL stimulation, we observed that ox-LDL decreased the viability of HUVECs (Figure 5a) and enhanced apoptosis (Figure 5b and C) of HUVECs. In addition, the protein and mRNA levels of the pro-apoptotic protein Bax were increased (Figure 5d and E), while the anti-apoptotic protein Bcl-2 decreased (Figure 5d and F). Additionally, we observed an increase in the secretion of progressive atherosclerosis cytokines, such as IL-6, IL-1β, and TNF-α, in the ox-LDL treated HUVEC supernatants (Figure 5g, H, I).

(A) The viability of HUVECs in the ox-LDL group was significantly decreased; (B–C). Apoptosis was significantly enhanced in the ox-LDL group; (D–F) Bax and Bcl-2 expression determined by Western blotting and qRT-PCR; (G–I) The secretion of cytokines in cell supernatants determined by ELISA kits. All experiments were repeated three times. **p < 0.01 vs. Control; ##*p* < 0.01 vs. ox-LDL+control-siRNA; &&*p* < 0.01 vs. ox-LDL+circCHFR-siRNA+inhibitor control.

Furthermore, compared with that in the ox-LDL+control-siRNA group, the viability of HUVECs in the ox-LDL+circCHFR-siRNA group was significantly improved (Figure 5a), and apoptosis was significantly reduced (Figure 5b and c). The protein and mRNA levels of Bax decreased (Figure 5d and E) whereas those of Bcl-2 increased (Figure 5d and F). TNF-α, IL-6, and IL-1β secretion was considerably reduced (Figure 5g, h, i,). However, the effects of circCHFR silencing were dramatically reversed by treatment with the miR-15b-5p inhibitor. These results suggest that the impact of circCHFR is dependent on miR-15b-5p expression.

### Regulatory effect of miR-15b-5p on GADD45G expression in HUVECs

**Figure 6.**
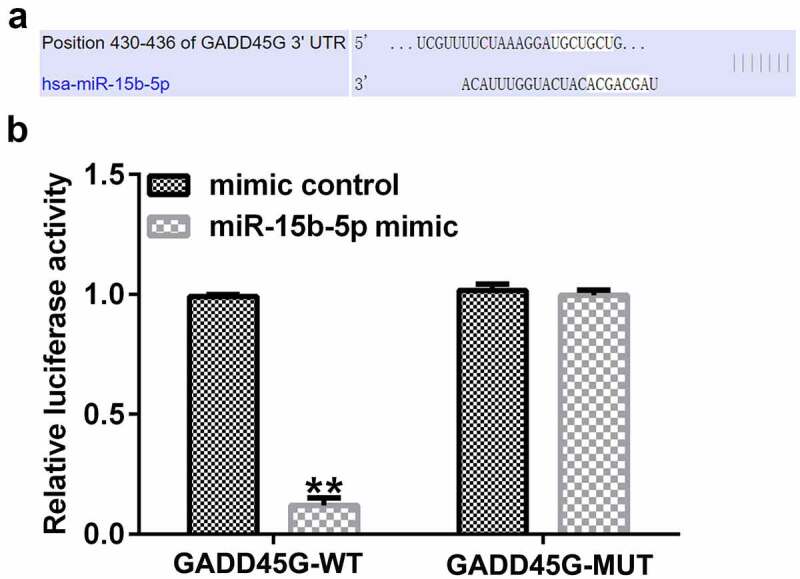
miR-15b-5p directly targets GADD45G.

**Figure 7.**
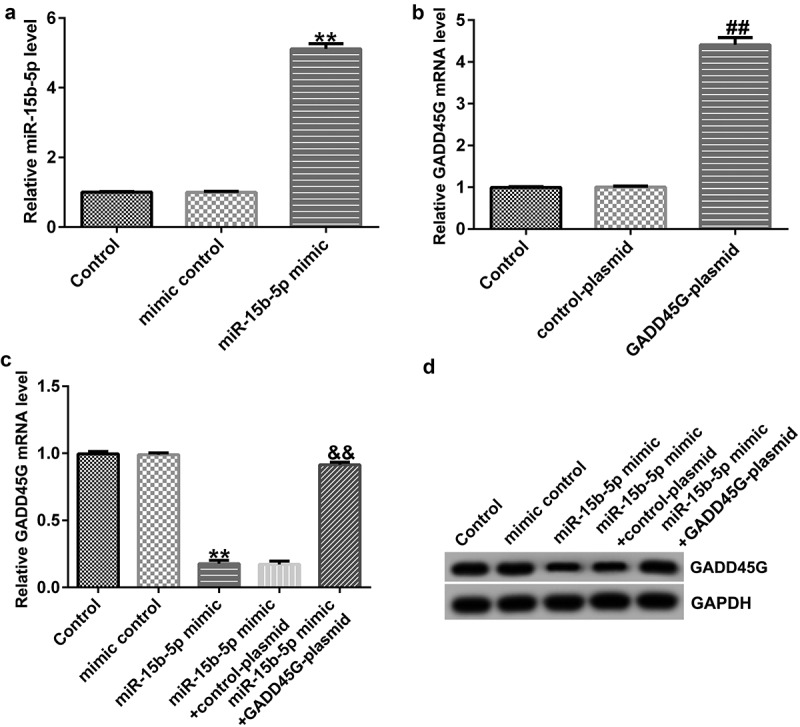
miR-15b-5p negatively regulates GADD45G expression in HUVECs.

To identify the binding sites of GADD45G and miR-15b-5p (Figure 6a), TargetScan was used. We verified the binding site using a dual fluorescent protein reporter system (Figure 6b). The role of miR-15b-5p in regulating GADD45G was further investigated by transfecting HUVECs with control plasmid or GADD45G plasmid, mimic control or miR-15b-5p mimic, and miR-15b-5p mimic+control plasmid or miR-15b-5p mimic+GADD45G plasmids. Transfection efficiency was evaluated using qRT-PCR. We discovered that the miR-15b-5p mimic significantly improved the expression of miR-15b-5p in HUVECs (Figure 7a), and GADD45G-plasmid increased the expression of GADD45G (Figure 7b). Moreover, the overexpression of miR-15b-5p significantly lowered GADD45G mRNA and protein expression in HUVECs, and these effects were reversed by GADD45G-plasmid co-transfection (Figure 7c and d).

(A) TargetScan predicting the miR-15b-5p and GADD45G interaction; (B) Dual luciferase assay verifying the binding location. All experiments were repeated three times. **p < 0.01 vs. mimic control

(A) Mimic control, and miR-15b-5p mimic were transfected into HUVECs, and then the level of miR-15b-5p was measured using RT-qPCR; (B) Control plasmid and GADD45G-plasmid were transfected into HUVECs, and then the mRNA level of GADD45G was measured using RT-qPCR; (C and D) miR-15b-5p mimic+control plasmid and miR-15b-5p mimic+GADD45 G plasmid were transfected into HUVECs, and then the mRNA and protein levels of GADD45 G were measured using RT-qPCR and Western blot assay. All experiments were repeated three times. **p < 0.01 vs. mimic control; ##*p* < 0.01 vs. Control-plasmid; &&*p* < 0.01 vs. miR-15b-5p mimic+control-plasmid.

### miR-15b-5p inhibits ox-LDL-induced HUVEC damage by downregulating GADD45G

**Figure 8.**
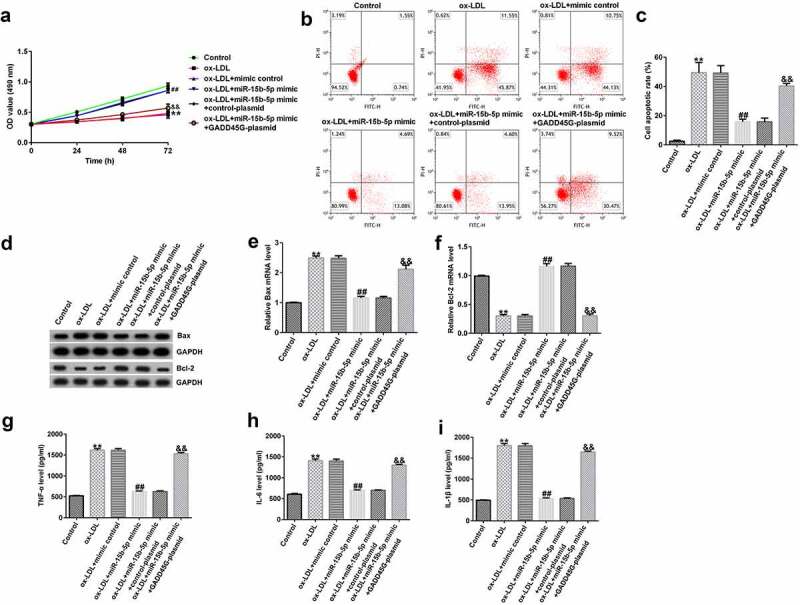
Protective role of miR-15b-5p in ox-LDL-treated HUVECs.

Finally, to further elucidate the role of miR-15b-5p in HUVECs injury, we transfected HUVECs with mimic control or miR-15b-5p mimic, and miR-15b-5p mimic+control plasmid or miR-15b-5p mimic+GADD45G-plasmid. Compared to the ox-LDL+mimic control group, we found that HUVEC viability in the ox-LDL+miR-15b-5p mimic group was significantly improved (Figure 8a), and apoptosis was significantly reduced (Figure 8b and C). The expression of Bax was downregulated (Figure 8d and E); however, the expression of Bcl-2 was significantly upregulated (Figure 8d and F). Also, the secretion of TNF-α, IL-6, and IL-1β was significantly reduced (Figure 8g, h, i). Further, these effects were reversed by the addition of GADD45G-plasmid. Therefore, the protective role of miR-15b-5p in atherosclerosis is directly associated with GADD45G.

(A) The cell viability of ox-LDL group; (B–C) Cell apoptosis detected by flow cytometry; (D–F) The levels of Bax and Bcl-2 determined by Western blotting and qRT-PCR; (G–I) Cytokine expression determined by ELISA kits. All experiments were repeated three times. **p < 0.01 vs. Control; ##*p* < 0.01 vs. ox-LDL+mimic control; &&*p* < 0.01 vs. ox-LDL+miR-15b-5p mimic+control-plasmid.

### Discussion

Atherosclerosis is linked to cardiovascular disorders, including peripheral arterial disease, myocardial infarction, CHD, stroke, and coronary artery disease [33]. In recent years, various medications have been introduced to manage atherosclerosis, which reduced morbidity and mortality in the developed countries, while the developing countries still experience high atherosclerosis-associated death rates [34]. Oxidative stress and high ox-LDL levels are major risk factors associated with atherosclerosis [35–37]. Ox-LDL-induced endothelial cell injury results in the development and progression of atherosclerosis [37]. Nonetheless, the underlying mechanism of ox-LDL-induced atherosclerosis remains largely unknown.

CircRNAs are dysregulated and abnormally expressed in angiogenesis-related diseases, such as atherosclerosis [38]. Yang et al evaluated the dysregulated circRNAs in VSMCs and found that circCHFR is one of the highly expressed circRNAs in ox-LDL-treated cells [16]. In this study, HUVECs were exposed to different doses of ox-LDL. Similarly, when HUVECs were exposed to ox-LDL induced stress, we observed a significant increase in circCHFR expression. Importantly, we observed significantly upregulated circCHFR expression in patients with atherosclerosis. However, we did not detect the expression of circCHFR in atherosclerotic plaque tissue, which was a limitation of this study. Previous studies have also demonstrated that circCHFR is upregulated in ox-LDL-challenged VSMCs, and regulates their proliferation, migration, and invasion [6,39]. This evidence suggests that circCHFR may play a role in atherosclerosis development. However, the exact mechanism by which circCHFR participates in the control of VSMC function remains unelucidated.

To further understand the involvement of circCHFR in ox-LDL-mediated cell death, the bioinformatics analysis tool starBase was used to predict the binding site; it identified miR-15b-5p as an important target of circCHFR. This was further validated using the luciferase reporter assay and the RNA pull-down method. miR-15b-5p exerts a protective role against atherosclerosis by downregulating apoptosis, oxidative stress, and inflammatory processes [40,41]. miR-15b-5p has also been reported to regulate coronary atherosclerotic heart disease progression through regulating endothelial progenitor cells autophagy by targeting MAPK1 [42]. Furthermore, we observed decreased miR-15b-5p levels in both ox-LDL-exposed HUVECs and patients with atherosclerosis. Our findings suggest that circCHFR directly regulates the expression of miR-15b-5p in HUVECs. The observed effects were reversed after miR-15b-5p inhibition, suggesting a strong interaction between circCHFR and miR-15b-5b.

The viability of HUVECs was drastically reduced in the ox-LDL group, while apoptosis was significantly increased. In addition, the pro-apoptotic protein Bax was elevated considerably, whereas the anti-apoptotic protein Bcl-2 was reduced. In addition, TNF-α, IL-6, and IL-1β proinflammatory cytokines were highly upregulated in the ox-LDL-treated group. These proinflammatory cytokines are involved in accelerating atherosclerosis progression; for example, IL-6 promotes cell adhesion and high fat-induced atherosclerosis [43,44].

On the contrary, silencing circCHFR in HUVECs significantly increased cell viability, whereas apoptosis was significantly reduced. The protein and mRNA levels of Bax were reduced, whereas those of Bcl-2 increased (Figure 5d and f). The secretion of TNF-α, IL-6, and IL-1β was significantly reduced. Moreover, the effects of circCHFR-siRNA were completely reversed by treatment with the miR-15b-5p inhibitor. Therefore, under ox-LDL stress, miR-15b-5p is a vital protector of HUVECs. However, the underlying molecular mechanism by which miR-15b-5p exerts a protective role against atherosclerosis is unknown.

To explore the molecular mechanism, we used TargetScan to explore the binding location of miR-15b-5p and GADD45G exhibited strong bonding, which was verified by a dual luciferase assay. GADD45G is a vital regulator of the stress response [22]. To confirm the miR-15b-5p and GADD45G relationship, we overexpressed miR-15b-5p and found that the miR-15b-5p mimic substantially reduced GADD45G mRNA and protein expression. Furthermore, the cell viability was enhanced, whereas, apoptosis was decreased in the miR-15b-5p mimic group; Bax expression downregulated significantly, while Bcl-2 expression upregulated significantly. Additionally, TNF-α, IL-6, and IL-1β secretion was significantly downregulated in the cell supernatant. However, these effects were reversed by GADD45G-plasmid. This suggests that miR-15b-5p protects HUVECs by downregulating GADD45G expression.

In summary, the present study indicated that circCHFR affect the ox-LDL-induced endothelial cells injury through the miR-15b-5p/GADD45G pathway (Supplementary Figure 1S).

## Conclusion

We found that the level of circCHFR was increased in atherosclerosis and ox-LDL-exposed endothelial cells. Silencing the circCHFR gene inhibited the ox-LDL-induced damage of endothelial cells via regulation of the miR-15b-5p/GADD45G axis. This study revealed a novel mechanism in atherosclerosis development and suggested that circCHFR could be a potential target in atherosclerosis management.

## Supplementary Material

Supplemental Material

## Data Availability

The datasets used and/or analyzed during the current study are available from the corresponding author on reasonable request.

## Research Highlights

- circCHFR was significantly upregulated, while miR-15b-5p was downregulated in the serum of atherosclerosis patients;
- circCHFR gene silencing protects against HUVEC injury via miR-15b-5p upregulation;
- miR-15b-5p inhibits ox-LDL-induced HUVEC damage by downregulating GADD45G.
