# Supplementary figures and images for: Circular RNA circCHFR downregulation protects against oxidized low-density lipoprotein-induced endothelial injury via regulation of microRNA-15b-5p/growth arrest and DNA damage inducible gamma
Source: Bioengineered. 2022 Feb 9;13(2):4481–92. doi: 10.1080/21655979.2022.2032967 (PMC8973773; doi:10.1080/21655979.2022.2032967)

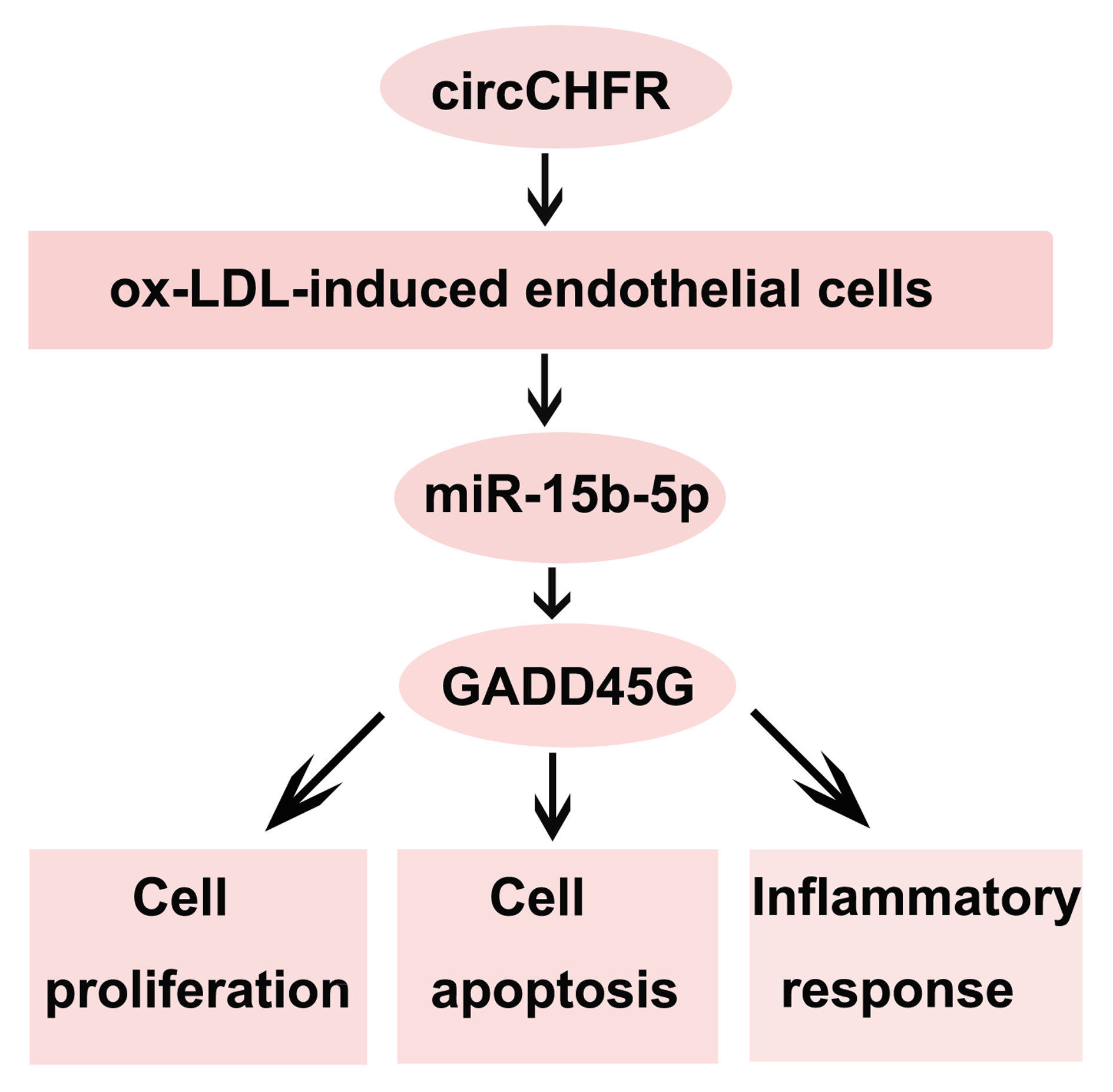

Supplement: Supplemental Material [file KBIE_A_2032967_SM4143.tif]
